# Ultrasonography Cannot Exclude Osteomyelitis in Diabetic Foot Ulcers: A Cross‐Sectional Diagnostic Accuracy Study Against Magnetic Resonance Imaging

**DOI:** 10.1002/edm2.70320

**Published:** 2026-09-04

**Authors:** Maryam Haghighi‐Morad, Hadi Vahedi, Arian Yousefi Fard, Behrooz Mohammadkhani Pordanjani, Seyyed Taher Seyyed Mahmoudi

**Affiliations:** ^1^ Department of Radiology Loghman Hakim Hospital, Shahid Beheshti University of Medical Sciences Tehran Iran; ^2^ Research Center for Evidence‐Based Health Management Maragheh University of Medical Sciences Maragheh Iran; ^3^ Department of Ophthalmology, Nikookari Eye Center Tabriz University of Medical Sciences Tabriz Iran; ^4^ Student Research Committee Tabriz University of Medical Sciences Tabriz Iran

**Keywords:** abscess, cellulitis, diabetic foot ulcer, diagnostic concordance, MRI, osteomyelitis, ultrasonography, wagner classification

## Abstract

**Background and Aims:**

Diabetic foot ulcers (DFUs) can lead to amputation, especially when complicated by osteomyelitis (OM) or soft tissue infection. Although MRI is the standard imaging method, ultrasonography (US) is increasingly used because it is more accessible. This study compared the diagnostic performance of US and MRI in DFU‐related infections.

**Methods:**

In this descriptive cross‐sectional study, consecutive adults with diabetes and suspected diabetic foot infection underwent both US and MRI. One ulcer per patient wasanalysed. US was compared with expert clinical assessment for cellulitis and with MRI for abscess and OM, following IWGDF recommendations. Diagnostic measures were calculated overall and by ulcer severity.

**Results:**

Among 127 patients, most had advanced Wagner grades (3–5) and forefoot ulcers. Cellulitis was clinically present in all cases; MRI and US concordance with the clinical diagnosis was 100.0% and 99.2%, respectively. For abscesses, US showed 100% sensitivity but low specificity, resulting in moderate accuracy. For OM, US had very poor sensitivity (17.9%) despite high specificity, especially in severe ulcers. MRI detected no OM in moderate‐grade ulcers.

**Conclusion:**

US showed near‐complete concordance with MRI for cellulitis, though this was assessed in a study in which cellulitis was universally present, and US performs poorly for OM and cannot reliably exclude bone infection. It is best used as an initial triage tool, while MRI remains essential when OM is suspected. Because MRI is not a perfect reference standard, the findings reflect diagnostic concordance rather than absolute accuracy and support a tiered imaging approach.

## Graphical abstract

Ultrasound is a valuable first‐line imaging tool for diabetic foot soft‐tissue infection but cannot reliably exclude osteomyelitis. MRI remains the preferred modality for evaluating suspected bone involvement.

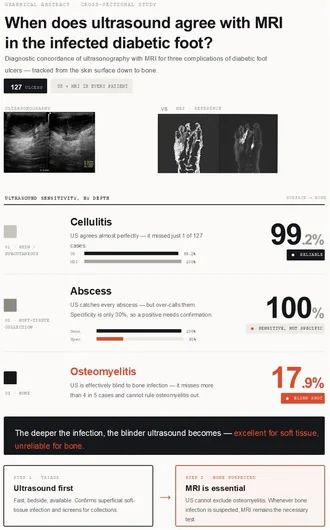

## 1 Introduction

As one of the most frequently recognised complications of poorly controlled and chronic diabetes, Diabetic foot ulcers (DFU) affect 19% to 34% of diabetic patients [1]. Inadequate glycemic regulation, peripheral vascular disease, and neuropathy are well‐established mechanisms underlying the development of DFU. Compared to non‐diabetics, patients with DFU have an 8‐fold higher risk of lower extremity amputation (LEA) [2]. Hence, imposing a Substantial Burden on the healthcare system, impairs physical function, and diminishes quality of life as numerous consequences of DFU [3].

One of the great contributors to amputation risk is osteomyelitis (OM). Observed in 10%–15% of moderate and 50% of severe infections, OM occurs due to the spread of juxtaposed soft tissue infection and penetration through the cortical bone into the medullary cavity [4, 5]. Thus, early diagnosis of OM and other soft tissue infections, such as abscesses, can prevent highly morbid outcomes of DFU.

Bone biopsy remains the definitive diagnostic modality for OM, integrating histopathological examination and bone culture to establish a conclusive diagnosis and treatment [6]. Nevertheless, the invasive nature of the procedure and its time‐intensive requirements often limit its feasibility in clinical practice [7]. As an alternative, imaging studies have emerged as the primary diagnostic approach for OM. Conventional radiography, specifically plain film radiography (PFR), can detect approximately 80% of OM cases in diabetic foot (DF) infections [8]. However, due to its delayed sensitivity in detecting osseous abnormalities—sometimes requiring 10‐20 days post‐infection—it is not the preferred initial diagnostic tool [9].

The Infectious Disease Society of America (IDSA) guidelines endorse magnetic resonance imaging (MRI) for diagnosing diabetic foot osteomyelitis (DFO), while simultaneously emphasising that bone biopsy, accompanied by pathological and microbiological analysis, remains the definitive diagnostic criterion [10]. With a pooled sensitivity of 0.90 (confidence interval 0.82–0.95), a specificity of 0.79 (confidence interval 0.62–0.91), and a diagnostic odds ratio of 24.4, MRI is considered to be the best imaging technique for the early diagnosis of DFO [11]. La Fontaine et al. also reported sensitivity and specificity of MRI improving to 90% and 74%, and the positive and negative predictive values rising to 88% and 78%, respectively, after a second MRI reading for DFO [12].

Despite limited recent publications, ultrasonography(US) remains an effective diagnostic tool among clinicians due to its procedural simplicity and widespread availability [13]. Although its sensitivity is inferior to that of MRI, limited comparative studies have evaluated the diagnostic efficacy of these two imaging techniques. This study seeks to compare MRI and US in diagnosing OM, as well as their utility in detecting associated soft tissue infections such as abscesses.

## 2 Materials and Methods

### 2.1 Study Design and Reporting Guideline

This was a single‐centre, descriptive‐analytical cross‐sectional study conducted between January 2024 and March 2025. The study was designed and reported in accordance with the Standards for Reporting of Diagnostic Accuracy Studies (STARD 2015) guidelines [14]. The objective was to evaluate the diagnostic performance of sonography compared with MRI for detecting cellulitis, abscess, and OM in patients with DFUs.

### 2.2 Study Setting

The study was performed at Loghman Hakim Hospital, a tertiary referral academic medical centre affiliated with Shahid Beheshti University of Medical Sciences, Tehran, Iran. This institution serves as a referral centre for patients with DF complications from both outpatient clinics and inpatient services. All imaging examinations were performed as part of routine clinical care in the radiology department.

### 2.3 Participants

Consecutive adult patients with diabetes mellitus and clinically suspected DF infection who were referred for imaging evaluation and underwent both sonography and MRI were considered for inclusion. Each patient contributed only one DFU to the analysis to avoid within‐patient clustering.

### 2.4 Inclusion Criteria

Patients were included if they met all of the following criteria:

- Age ≥ 18 years
- Confirmed diagnosis of diabetes mellitus
- Presence of a clinically suspected DFU ulcer, based on clinical examination by the treating physician
- Referral for both ultrasound and MRI examination as part of routine diagnostic evaluation
- Availability of complete imaging and clinical data

### 2.5 Exclusion Criteria

Patients were excluded if they met any of the following criteria:

- Foot infection unrelated to diabetes mellitus
- Prior surgical intervention at the same site before imaging
- Incomplete imaging studies or non‐diagnostic image quality
- Absence of either ultrasound or MRI examination
- Refusal to participate or incomplete medical records
- Inability to undergo contrast‐enhanced MRI, including estimated glomerular filtration rate below 30 mL/min/1.73 m^2^, known hypersensitivity to gadolinium‐based contrast agents, or patient refusal of contrast administration
- Indeterminate MRI bone findings, defined as fluid‐sensitive hyperintensity involving cortical bone without confluent T1 marrow replacement (a pattern described as “osteitis”), which could not be confidently assigned to either the presence or absence of osteomyelitis

### 2.6 Ethical Approval and Informed Consent

This study was approved by the Ethics Committee of Shahid Beheshti University of Medical Sciences (IR.SBMU.MSP.REC.1403.069) and conducted in accordance with the Declaration of Helsinki. Written informed consent was obtained from all participants before inclusion.

### 2.7 Clinical and Ulcer Assessment

Recent guidelines acknowledge the continued clinical utility of the Wagner classification system, particularly in resource‐limited settings such as our institution, yet emphasise its limitations in diagnostic and staging precision for DF infections when compared to more comprehensive systems like SINBAD [15]. While the Wagner system remains widely implemented due to its relative feasibility, its insufficient infection‐specific granularity has led to its exclusion from recommendations for the assessment and classification of DF infections.

Ulcers were graded according to the Wagner–Meggitt classification system as follows:

- Grade 0: Intact skin with pain only.
- Grade 1: Superficial and localised ulcer of skin or subcutaneous tissue.
- Grade 2: Deep ulcer reaching tendon, bone, or joint capsule.
- Grade 3: Deep ulcer with bone involvement or abscess.
- Grade 4: Gangrene of toes or forefoot.
- Grade 5: Midfoot or hindfoot gangrene (full‐foot gangrene).

For analysis, ulcers were categorised into two severity groups: Moderate ulcers, including Wagner grades 1–2, and severe ulcers consisting of grades 3–5.

### 2.8 Ultrasound and MRI Examination

Magnetic resonance imaging was performed using a 1.5 T Magnetom Amira SiemensCorp. device. Each patient underwent pre‐contrast imaging in T1w, T2w, PD fat sat, DWI and ADC sequences and post‐contrast imaging with T1 fat sat sequences. All of these sequences were performed in axial, coronal and sagittal planes except DWI and ADC, which were done in the axial plane with b values: 400 and 800. The other imaging parameters were: Slice thickness: 3.5 mm, matrix: 352*240, FOV: 230*230. Ultrasound images were performed using a Supersonic MACH30 device and an L18‐5 transducer (5–18 MHz).

**FIGURE 1:**
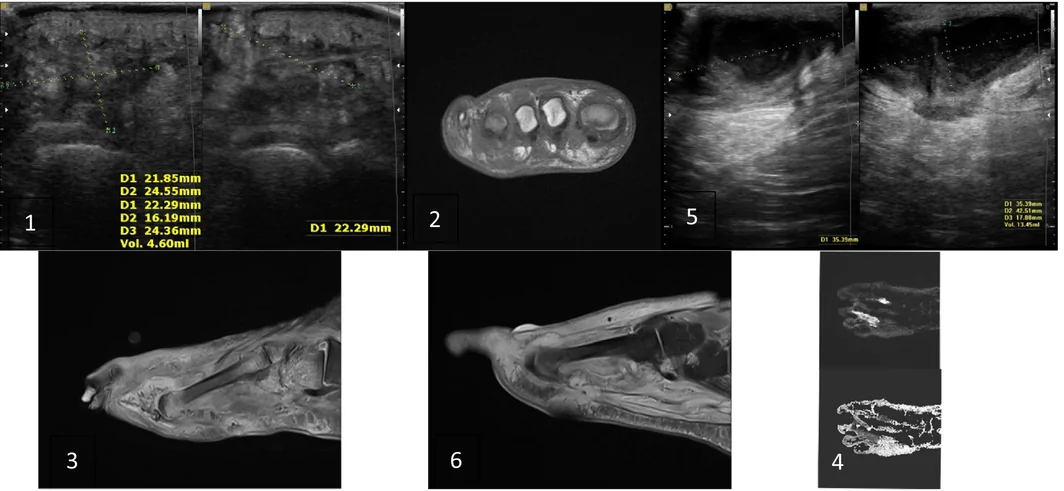
Multimodal imaging of DF infection in the same patients. (1) Ultrasound demonstrates a hypoechoic soft tissue collection consistent with an abscess. (2) Axial T1‐weighted MRI reveals OM involving the fourth metatarsal head. (3) Sagittal proton density (PD) sequence shows a plantar skin defect at the metatarsophalangeal (MTP) joint with an adjacent soft tissue collection. (4) Axial diffusion‐weighted imaging (DWI, *b* = 800) and corresponding apparent diffusion coefficient (ADC) map highlight a diffusion‐restricted collection in the forefoot, suggestive of an abscess. (5) There is increased echogenicity of subcutaneous fat and fluid separating fat lobules, suggestive of cellulitis on US. Also, a fluid collection (abscess) is seen. (6) Sagittal PD fat‐sup MRI of the foot revealed OM in the head of the 3rd metatarsal bone, surrounding an air‐containing collection.

The diagnostic criteria for OM, abscess, and cellulitis were established based on MRI and sonographic findings. For OM [16], MRI findings included bone marrow oedema, which appears as a low signal intensity on T1‐weighted images and a high signal intensity on T2‐weighted or fluid‐sensitive sequences and DWI restriction, along with cortical bone destruction marked by the loss of the normal cortical bone margin adjacent to the skin ulcer and resultant cellulitis. Any type of bony signal change further from the skin ulcer is accepted as bone marrow oedema or neuropathic arthritis. Sonographic criteria for OM comprised periosteal elevation or thickening, subperiosteal abscess formation, and cortical erosions with direct fluid contact with the bone, all of which were considered highly indicative of the condition. For abscess diagnosis [17], MRI criteria involved a well‐defined fluid collection exhibiting low signal intensity on T1‐weighted images and high signal intensity on T2‐weighted images, DWI restriction and peripheral rim enhancement following contrast administration with surrounding inflammatory changes. On sonography, abscesses were identified as anechoic or hypoechoic collections with posterior acoustic enhancement, irregular borders, and occasionally observable pus movement. In the case of cellulitis [18], MRI findings included subcutaneous oedema, skin and soft tissue thickening, and the absence of a discrete fluid collection—key features distinguishing it from an abscess. Cellulitis is diagnosed when the area of soft tissue signal change is in close proximity to a skin ulcer and shows contrast enhancement; however, Stasis oedema is accepted in cases with more diffuse signal change and without a skin ulcer or clinical signs of cellulitis. Sonographic evaluation of cellulitis revealed a characteristic “cobblestone” appearance due to subcutaneous oedema, increased echogenicity, and indistinct tissue borders. You can see the multimodal imaging of a DF infection for the same patients in Figure 1.

Ultrasound examinations were performed and interpreted by a single radiologist with approximately 10 years of post‐qualification experience, whereas MRI examinations were interpreted independently by a different radiologist with approximately 15 years of post‐qualification experience. No second reader was available for either modality, and each examination was interpreted by a single assessor without consensus review. Ultrasonography was performed first in all participants, followed by MRI after a median interval of 2 days; therefore, ultrasound interpretation was completed without knowledge of the MRI findings. The MRI reader was blind to the ultrasound images and report during interpretation. Both readers had access only to the routine clinical information available at the time of imaging (including age, sex, diabetes status, ulcer site, and the clinical indication for imaging), reflecting routine clinical practice. All interpretations were finalised and recorded independently before the paired imaging data were compared for analysis.

Indeterminate findings were handled by exclusion rather than by forced classification, so that every analysed case reflected a confident interpretation.

### 2.9 Reference Standards

Diagnosing complications in diabetic foot infections presents inherent methodological challenges, as subjecting every study participant to a definitive, invasive gold standard (such as bone biopsy or surgical exploration) is not ethically justifiable. To address this limitation, and in accordance with international guidelines (IWGDF/IDSA), we defined our reference standards using the best available, non‐invasive modalities.

For OM and deep abscesses, MRI was performed on all patients and served as the reference standard. MRI is currently recommended by the IWGDF as the most accurate advanced imaging modality for mapping deep soft tissue infections and bone involvement in the diabetic foot. By utilising MRI universally, we avoided the verification bias that occurs when only severely ill patients receive a reference test. The reference standard for cellulitis was the clinical diagnosis recorded by an infectious diseases specialist on the basis of local inflammatory signs (erythema, warmth, tenderness, and induration extending beyond the ulcer margin), applying IWGDF/IDSA criteria for soft‐tissue infection [19]. This assessment was made at the time of clinical presentation and before either imaging examination was performed.

### 2.10 Sample Size Calculation and Statistical Analysis

A precision‐based sample size calculation for diagnostic accuracy studies was performed using the Buderer method, with OM defined as the primary target condition [14]. Expected ultrasound sensitivity and specificity for OM were set at 79% and 80%, respectively, based on prior published evidence [20]. An expected OM prevalence of 60% in the study population, a 90% confidence level (Z=1.645), and a prespecified confidence interval half‐width (precision) of ±10% were used for the calculation. The required total sample size was estimated separately for sensitivity and specificity using Buderer's formulas, yielding 75 participants for sensitivity and 109 participants for specificity. The larger value (109 participants) was selected as the target sample size. Subgroup analyses according to ulcer severity (moderate vs. severe Wagner groups) were not included in the sample size determination and were therefore considered exploratory.

Descriptive statistics were used to summarise the demographic, clinical, and microbiological characteristics of the study population. Categorical variables were reported as frequencies and percentages, while continuous variables (e.g., age) were presented as medians and interquartile ranges (IQR) because their distribution was non‐normal, as assessed visually and by the Shapiro–Wilk test.

Diagnostic performance of sonography for detecting abscess and osteomyelitis was evaluated using 2 × 2 contingency tables, with MRI as the reference standard. Sensitivity, specificity, positive predictive value (PPV), negative predictive value (NPV), and overall accuracy were calculated using standard formulas, with corresponding 95% confidence intervals (CIs) estimated by the exact binomial (Clopper–Pearson) method in R (R Foundation for Statistical Computing, Vienna, Austria). Cellulitis was analysed separately because the clinical diagnosis of soft‐tissue infection constituted the indication for imaging referral; consequently, all participants were clinically positive, and no clinically negative cases were available. Under these circumstances, conventional diagnostic accuracy measures (sensitivity, specificity, PPV, NPV, and accuracy) are not estimable or clinically meaningful. Therefore, cellulitis findings are reported as positive per cent agreement (PPA) of ultrasound and MRI with the clinical diagnosis, as well as the agreement between the two imaging modalities, each with exact binomial 95% CIs. When a performance metric was not estimable because its denominator was zero, it was reported as not applicable (N/A).

Subgroup analyses according to ulcer severity (moderate vs. severe Wagner groups) were considered exploratory and were not used for sample size determination. Descriptive and primary diagnostic performance analyses were conducted using IBM SPSS Statistics version 26 (IBM Corp., Armonk, NY, USA).

## 3 Results

**FIGURE 2:**
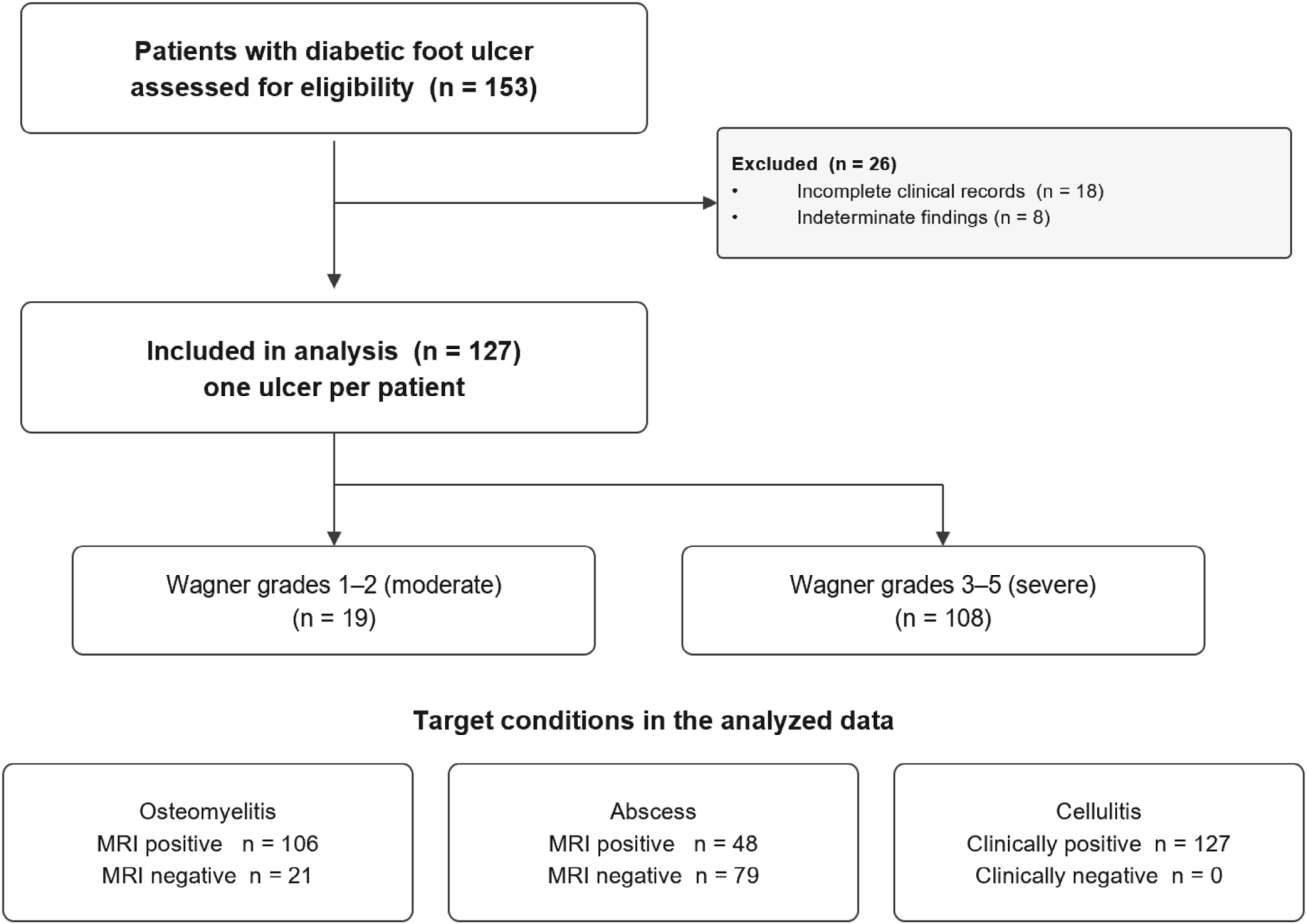
Participant flow diagram and distribution of target conditions.

A total of 153 patients with diabetic foot ulcers were assessed for eligibility, of whom 127 met the inclusion criteria and were included in the final analysis. Twenty‐six patients were excluded because of incomplete clinical records (*n* = 18) or indeterminate imaging findings (*n* = 8). The participant selection process and distribution of the target conditions are summarised in Figure 2.

The study consisted of 85 males (66.9%) and 42 females (33.1%), with a median age of 55 years (IQR: 46–62) and an age range between 38 and 85 years. According to the Wagner classification (Table 1), 37% of ulcers were classified as grade 3, 31.5% as grade 4, and 16.5% as grade 5, while only 6.3% and 8.7% were identified as grades 1 and 2, respectively. Anatomically, the most frequently involved zone was the forefoot (48%), followed by combined forefoot and midfoot involvement (35.4%). In contrast, isolated midfoot and hindfoot involvement were each present in only 3.1% of cases. The most common surface involvement was plantar (40.9%), followed by dorsal (33.1%), and combined dorsal and plantar (26%).

**TABLE 1:** Demographic and clinical characteristics of patients with DFUs.

Characteristic	Statistic
Sex, *n* (%)	
Male	85 (66.9%)
Female	42 (33.1%)
Age (Years)	
Median (IQR)	55 (46–62)
Range (min, max)	47 (38, 85)
Wagner grade, *n* (%)	
Grade 1	8 (6.3%)
Grade 2	11 (8.7%)
Grade 3	47 (37%)
Grade 4	40 (31.5%)
Grade 5	21 (16.5%)
Anatomic involvement zones of the foot, *n* (%)	
Fore foot	61 (48%)
Mid foot	4 (3.1%)
Hind foot	4 (3.1%)
Fore and midfoot	45 (35.4%)
Fore and Hind foot	0 (0%)
Mid and Hind foot	2 (1.6%)
Fore, Mid, and Hind foot	11 (8.7%)
Topographic involvement zones of the foot, *n* (%)	
Dorsum of the foot	42 (33.1%)
Plantar of the foot	52 (40.9%)
Dorsal and Plantar of the foot	33 (26%)

Among the 127 analysed participants, MRI identified osteomyelitis in 106 (83.5%). Prevalence differed markedly by ulcer severity: 0 of 19 ulcers (0%) in the moderate group (Wagner grades 1–2) and 106 of 108 (98.1%) in the severe group (Wagner grades 3–5). MRI‐defined abscess was present in 48 of 127 (37.8%): 2 of 19 (10.5%) moderate and 46 of 108 (42.6%) severe ulcers.

No imaging‐related complications or adverse events attributable to either examination were recorded.

Microbiological findings identified *Staphylococcus aureus* as the most prevalent pathogen (25.2%), followed by *Escherichia coli* (18.1%), *Staphylococcus epidermidis* (14.2%), Enterococcus spp. (13.4%), *Pseudomonas aeruginosa* (9.4%), *Klebsiella pneumoniae* (8.7%), and *Acinetobacter baumannii* (7.9%) (Table 1).

Cellulitis was clinically diagnosed in all 127 participants (100%), reflecting the referral pathway through which participants entered the study. MRI identified cellulitis in all 127 cases, giving a positive per cent agreement with clinical assessment of 100.0% (95% CI 97.1–100.0) (Table 2). Sonography identified cellulitis in 126 of 127 cases, a positive per cent agreement of 99.2% (95% CI 95.7–100.0) (Table 3). The two imaging modalities agreed in 126 of 127 cases (99.2%; 95% CI 95.7–100.0), the single discordant case being one in which sonography did not demonstrate the subcutaneous changes seen on MRI. Because no clinically negative cases were available, specificity, negative predictive value and overall accuracy for cellulitis are not estimable, and these concordance figures should not be read as measures of diagnostic accuracy: They describe how often each modality detected a condition already known to be present, not the ability of either modality to discriminate cellulitis from its absence.

**TABLE 2:** Concordance between MRI and clinical diagnosis of cellulitis.

Cellulitis		Clinical assessment	
		Negative	Positive
MRI	Negative	0	0
	Positive	0	127

**TABLE 3:** Concordance between sonography and clinical diagnosis of cellulitis.

Cellulitis		Radiopaedia.org clinical assessment	
		Negative	Positive
Sonography	Negative	0	1
	Positive	0	126

Diagnostic performance of sonography for detecting abscess demonstrated high sensitivity overall, reaching 100.0% (95% CI 92.6–100.0), but specificity was relatively low at 30.4% (95% CI 20.5–41.8), resulting in an overall accuracy of 56.7% (95% CI 47.6–65.5) (Table 4 and Table 6). Subgroup analysis showed that in moderate ulcers, sonography maintained a sensitivity of 100.0% (95% CI 15.8–100.0) and specificity of 58.8% (95% CI 32.9–81.6), although the PPV was low at 22.2% (95% CI 2.8–60.0). Despite this, the NPV was 100.0% (95% CI 69.2–100.0), and overall accuracy was 63.2% (95% CI 38.4–83.7). In severe ulcers, sensitivity remained 100.0% (95% CI 92.3–100.0), but specificity decreased to 22.6% (95% CI 12.9–35.0), with a PPV of 48.9% (95% CI 38.5–59.5) and NPV of 100.0% (95% CI 76.8–100.0). The overall accuracy in severe ulcers was 55.6% (95% CI 45.7–65.1), reflecting the higher number of false‐positive sonographic findings in this subgroup (Table 7).

**TABLE 4:** Comparison of sonography and MRI (imperfect reference standard) in detecting abscess.

Abscess		Sonography					
		Negative	Positive	Ulcer severity		Negative	Positive
MRI	Negative	24	55	Moderate Ulcers	Negative	10	7
					Positive	0	2
	Positive	0	48	Severe Ulcers	Negative	14	48
					Positive	0	46

Diagnostic performance of sonography for detecting OM was limited overall, with a sensitivity of 17.9% (95% CI 11.2–26.6) and an overall accuracy of 30.7% (95% CI 22.8–39.5), although specificity remained high at 95.2% (95% CI 76.2–99.9) (Table 5 and Table 6). In subgroup analysis, no cases of OM were identified by MRI in moderate ulcers, resulting in a specificity, NPV, and overall accuracy of 100.0% (95% CI 82.4–100.0), while sensitivity and PPV were not applicable due to the absence of MRI‐positive cases. In severe ulcers, sonography demonstrated poor sensitivity of 17.9% (95% CI 11.2–26.6), indicating a high number of false‐negative findings. Specificity in this subgroup was 50.0% (95% CI 1.3–98.7), while PPV remained high at 95.0% (95% CI 75.1–99.9). However, the NPV was extremely low at 1.1% (95% CI 0.0–6.2), and overall accuracy was only 18.5% (95% CI 11.7–27.1), highlighting the limited ability of sonography to reliably exclude OM in patients with severe DFUs (Table 7).

**TABLE 5:** Comparison of sonography and MRI (imperfect reference standard) in detecting OM.

Osteomyelitis		Sonography					
		Negative	Positive	Ulcer severity		Negative	Positive
MRI	Negative	20	1	Moderate Ulcers	Negative	19	0
					Positive	0	0
	Positive	87	19	Severe Ulcers	Negative	1	1
					Positive	87	19

**TABLE 6:** Overall diagnostic performance of sonography for detecting abscess and OM, using MRI as the imperfect reference standard.

Condition	Sensitivity % (95% CI)	Specificity % (95% CI)	PPV % (95% CI)	NPV % (95% CI)	Accuracy % (95% CI)
Abscess	100.0 (92.6–100.0)	30.4 (20.5–41.8)	46.6 (36.7–56.7)	100.0 (85.8–100.0)	56.7 (47.6–65.5)
Osteomyelitis	17.9 (11.2–26.6)	95.2 (76.2–99.9)	95.0 (75.1–99.9)	18.7 (11.8–27.4)	30.7 (22.8–39.5)

**TABLE 7:** Diagnostic performance of sonography according to ulcer severity.

Condition	Ulcer severity	Sensitivity % (95% CI)	Specificity % (95% CI)	PPV % (95% CI)	NPV % (95% CI)	Accuracy % (95% CI)
Abscess	Moderate	100.0 (15.8–100.0)	58.8 (32.9–81.6)	22.2 (2.8–60.0)	100.0 (69.2–100.0)	63.2 (38.4–83.7)
Abscess	Severe	100.0 (92.3–100.0)	22.6 (12.9–35.0)	48.9 (38.5–59.5)	100.0 (76.8–100.0)	55.6 (45.7–65.1)
Osteomyelitis	Moderate	*N*/A	100.0 (82.4–100.0)	*N*/A	100.0 (82.4–100.0)	100.0 (82.4–100.0)
Osteomyelitis	Severe	17.9 (11.2–26.6)	50.0 (1.3–98.7)	95.0 (75.1–99.9)	1.1 (0.0–6.2)	18.5 (11.7–27.1)

*Note:* N/A indicates metrics that could not be calculated because the denominator was zero.

## 4 Discussion

This study evaluated the diagnostic performance of sonography compared to MRI and clinical assessment for key complications of diabetic foot ulcers. The findings reveal a distinct pattern: While sonography demonstrates strong utility for detecting superficial soft‐tissue infections like cellulitis and abscess, its performance is markedly limited for diagnosing osteomyelitis. This discrepancy underscores the modality's potential role as an initial screening tool for soft tissue pathology but highlights its critical inadequacy in assessing bone involvement.

Diabetic foot infection represents a complex clinical entity in which imaging findings must be interpreted within a broader diagnostic framework that integrates clinical, laboratory, and microbiological data. Contemporary evidence‐based guidelines emphasise a stepwise approach, beginning with clinical assessment and plain radiography, followed by advanced imaging when diagnostic uncertainty persists [21, 22].

Contrast‐enhanced imaging is central to separating infective cellulitis from non‐infective interstitial or stasis oedema, since both produce fat reticulation and hyperintensity on fluid‐sensitive sequences, whereas enhancement of the involved subcutaneous tissue favours cellulitis; it is likewise central to the identification of abscess, for which peripheral rim enhancement is a defining criterion. For this reason, we required contrast‐enhanced imaging in every participant and excluded patients in whom it could not be performed, rather than applying different diagnostic criteria to different subsets of the study.

### 4.1 Ultrasound as an Initial Imaging Tool

Ultrasound is a valuable initial imaging modality for assessing cellulitis and soft tissue abscesses due to its accessibility and high sensitivity for certain findings [23, 24]. Normal subcutaneous tissue appears hypoechoic on ultrasound, with sparse hyperechoic connective tissue strands. Cellulitis appears as thickened subcutaneous tissue with increased echogenicity and a blurred look, progressing to oedema with striation and a “cobblestone” pattern [25]. Abscesses or joint effusions present as anechoic or hypoechoic spherical collections with enhanced through‐transmission, often featuring an echogenic capsule or septa in abscesses. Moreover, ultrasound surpasses MRI in detecting foreign bodies, particularly wooden ones, due to its superior sensitivity in this context [26]. However, ultrasound's operator‐dependent nature and variable probe quality may limit its consistency, necessitating comparison with more definitive modalities like MRI [27]. Our results highlight this ‘diagnostic ceiling,’ particularly regarding bone involvement. While the high sensitivity of ultrasound makes it a reliable tool for ruling out soft tissue abscesses, its failure to detect bone marrow changes (evidenced by our poor 17.9% sensitivity for OM) confirms that it cannot visualise early medullary or cortical changes with the precision of MRI. Current IWGDF(2023) guidelines recommend plain radiography as the first‐line imaging test, followed by MRI if OM is suspected but not confirmed [19].

These data dictate a precise clinical utility for ultrasonography within the diagnostic triage of diabetic foot ulcers. Rather than a substitute for advanced imaging, ultrasound functions as a high‐specificity ‘Rule‐In’ tool for soft‐tissue complications. Its primary value lies in the immediate bedside identification of abscesses, where its diagnostic performance justifies rapid surgical or percutaneous intervention without the delays inherent to MRI scheduling. However, the clinical utility of ultrasound reaches a definitive boundary regarding bone involvement. The 17.9% sensitivity for osteomyelitis, a finding supported by a subgroup analysis that met our predefined power requirements, confirms that a negative ultrasound lacks the negative predictive power to exclude bone infection. Consequently, we advocate for a diagnostic protocol where ultrasound facilitates rapid soft‐tissue triage, but where MRI remains a mandatory reflex investigation for any patient where osteomyelitis is clinically suspected but not visualised on ultrasound. This tiered approach optimises surgical timing for soft‐tissue infections while maintaining the necessary diagnostic rigour to prevent missed osteomyelitis and subsequent amputation.

### 4.2 Ultrasound Concordance for Cellulitis, and Diagnostic Performance for Abscess

In our research, Ultrasonography exhibited highly effective sensitivity of 99.2% (95% CI 95.7–100.0) concordance with the clinical diagnosis of cellulitis, a figure that reflects detection in a universally affected study rather than discriminative accuracy. Furthermore, our study indicated that ultrasonography achieved 100% sensitivity for the detection of abscesses in both moderate and severe ulcers, indicating a high ability to identify true positives. However, its specificity was limited (58.8% in moderate ulcers and 22.6% in severe ulcers), resulting in a high rate of false positives, reflected in the relatively low PPV (22.2% and 48.9%, respectively). These results suggest that while a negative sonographic study effectively rules out an abscess, a positive result does not reliably confirm a drainable collection. The high rate of false positives significantly undermines its utility in clinical decision‐making, especially when determining whether surgical drainage is warranted. As a result, our findings indicate that the US, in its current application, may be more useful as a rule‐out tool rather than for confirming the abscess.

In previous studies, Ultrasound has been shown to have a sensitivity of 97% and specificity of 83% in the diagnosis of abscess in soft tissue infection [28]. The higher diagnostic accuracy reported in these studies relative to our results may be influenced by factors such as abscess characteristics, variations in ultrasound equipment, and the inherent operator dependency of sonographic assessments.

In contrast, MRI remains the most comprehensive and sensitive tool for diagnosing soft tissue pathologies due to its superior tissue characterisation [29]. Calluses appear as focal subcutaneous prominences with low T1‐weighted (T1w) and low‐to‐intermediate T2‐weighted (T2w) signal intensity, occasionally associated with a bursa exhibiting fluid‐like characteristics (low T1w, high T2w) but lacking soft tissue reaction or contrast enhancement, distinguishing it from an abscess [23]. Soft tissue oedema and cellulitis present as fat reticulation with intermediate T1w and high T2w signal, with cellulitis demonstrating post‐contrast enhancement. Another distinguishing feature of cellulitis is its focal appearance and close proximity to a skin ulcer compared to the more diffuse nature of stasis oedema. Abscesses appear as fluid collections with rim enhancement post‐contrast, though similar findings may occur in haematomas ortumours, necessitating careful differential diagnosis [30].

### 4.3 Ultrasound Limitations in Osteomyelitis Diagnosis

There are various findings on ultrasound indicating the presence of OM. Bone cortex irregularity, characterised by the loss of the normal smooth hyperechoic cortical line, suggests erosive changes, consistent with bone infection. Moreover, a subperiosteal abscess appears as a hypoechoic fluid collection beneath the periosteum, further supporting the diagnosis [31]. Surrounding soft tissue oedema, demonstrated by hyperechogenicity compared to normal tissue, indicates associated infection. The presence of both cortical irregularity and a subperiosteal abscess strongly suggests an OM diagnosis [32, 33, 34].

In our study, ultrasound exhibited poor diagnostic accuracy for OM in higher‐grade DFUs (Wagner grades 3–5), with a sensitivity of 17.9% and specificity of 50%, reflecting a high rate of missed diagnoses. This strikingly low sensitivity is a finding of paramount clinical importance, as it quantifies the risk of ‘false reassurance.’ The resulting wide confidence intervals for certain metrics necessitate a conservative diagnostic approach; specifically, they confirm that while ultrasonography provides utility in identifying soft‐tissue collections, it lacks the diagnostic power to exclude bone involvement. Consequently, relying on a negative ultrasound to ‘rule out’ osteomyelitis poses a substantial risk of delayed diagnosis. In such cases, the escalation to MRI remains a clinical necessity to mitigate the risk of inadequate debridement and subsequent amputation.

Few studies have examined ultrasound accuracy in the diagnosis of OM in DFU. Due to operator dependence and probequality, previous studies have reported higher performance. Enderle et al. conducted a study evaluating ultrasound diagnostic performance in detecting chronic OM, reporting a sensitivity of 78.6% (49.2–95.3) and specificity of 80% (28.4–99.5) [20]. The wide confidence intervals demonstrated in their findings indicate inconsistency in ultrasound's reliability, consistent with our study's observation of limited diagnostic accuracy for OM in higher‐grade DFUs (Wagner grades 3–5).

Other studies have also assessed the role of ultrasound in detecting OM among non‐diabetic populations. One of these studies were done by Hosokawa et al. to evaluate the diagnostic accuracy of ultrasound in paediatric OM cases, demonstrating a NPV of 73.3% (11/15) (95% CI 44.9–92.2), a PPV of 100% (8/8), (95% CI 63.1–100), sensitivity of 66.7% (8/12) (95% CI 34.9–90.1), specificity of 100% (11/11) (95% CI 71.5–100), positive likelihood ratio of > 99, and a negative likelihood ratio of 0.33, respectively [34]. Hosokawa cautioned that while sonography may be helpful, its diagnosis can be misleading. False interpretation may arise due to several factors, such as the absence of bone cortex irregularity in early OM and the operator‐dependent nature of subperiosteal abscess, which could be challenging in deep abscesses. Riebel et al. concluded that in acute haematogenous OM, ultrasound can detect abnormalities within 1–2 days of symptom onset, provided the affected area is accessible (e.g., extremities, hips, ribs, clavicle). Hence, sonography is proposed as a helpful alternative to MRI for early diagnosis of OM in these locations [35, 36].

### 4.4 Limitations and Future Directions

The primary methodological consideration and limitation of this study is the use of imperfect reference standards, a common and often unavoidable challenge in diabetic foot research. Specifically, MRI was used as the reference standard for osteomyelitis and deep abscess rather than the definitive gold standard of bone biopsy or surgical exploration. While MRI is the most accurate non‐invasive imaging modality and is recommended by international guidelines for clinical practice, it is not infallible. Specifically, Charcot neuroarthropathy remains a significant diagnostic challenge, as the reactive bone marrow oedema and joint destruction seen in the active phase of Charcot can closely mimic the signal characteristics of osteomyelitis. This overlap may lead to reduced specificity in the reference standard itself, a factor that should be considered when interpreting the concordance between US and MRI findings [21, 37].

Additionally, the diagnostic performance of ultrasound is constrained by operator dependency and equipment variability, which may explain discrepancies between our findings and those of prior studies. Furthermore, each imaging modality was interpreted by a single experienced radiologist, and formal assessment of intra‐ or interobserver reproducibility was not performed because no second reader was available during the study period. Consequently, the reported diagnostic performance reflects the performance of one experienced reader per modality at a single tertiary centre rather than the average performance achievable across readers. Given the operator‐dependent nature of ultrasonography, the reproducibility of these findings in other settings remains uncertain, and future prospective multi‐reader studies incorporating formal agreement analyses are warranted before these results can begeneralised.

In addition, requiring contrast‐enhanced MRI excluded patients with advanced renal impairment. Chronic kidney disease is common in this population and is associated with more severe foot disease and higher amputation risk, so our findings may not extend to that subgroup—a group in whom non‐contrast MRI or ultrasound may in practice be the only available options, and in whom the diagnostic performance of ultrasound therefore matters most. Also, patients with indeterminate MRI bone findings were excluded rather than adjudicated. This “grey zone” exclusion improves the internal consistency of the reference standard but restricts the applicability of our estimates to patients with unambiguous imaging findings, and it is a recognised source of optimistic bias in diagnostic accuracy studies.

Furthermore, our study's sample size and focus on DFUs limit generalisability to other soft tissue pathologies and the predominance of severe ulcers in our cohort introduces spectrum bias, potentially limiting applicability to patients with milder disease. To address statistical instability in subgroup analyses, future studies should prospectively stratify patients by ulcer severity with dedicated sample sizes for each subgroup. It is also recommended to standardise ultrasound protocols to enhance reproducibility and compare the cost‐effectiveness of ultrasound vs. MRI in resource‐limited settings.

## 5 Conclusion

This study demonstrates that while sonography exhibits high sensitivity for detecting superficial soft‐tissue complications of diabetic foot ulcers (cellulitis and abscess), it performs poorly for diagnosing osteomyelitis. The modality's low sensitivity and negative predictive value for bone infection highlight its limitations as a standalone diagnostic tool for osteomyelitis, despite its utility in initial soft‐tissue assessment. These findings underscore the importance of complementary MRI for comprehensive evaluation of bone involvement in diabetic foot infections. Importantly, the results reflect diagnostic concordance with MRI—acknowledged as an imperfect reference standard—rather than absolute accuracy against histopathology.

## Author Contributions

**Maryam Haghighi‐Morad:** conceptualization, supervision, project administration, writing – review and editing. **Arian Yousefi Fard:** conceptualization, project administration. **Hadi Vahedi:** conceptualization, methodology, data curation, supervision, formal analysis, writing – review and editing, writing – original draft, visualization. **Behrooz Mohammadkhani Pordanjani:** conceptualization, project administration. **Seyyed Taher Seyyed Mahmoudi:** conceptualization, writing – original draft.

## Funding

The authors have nothing to report.

## Disclosure

Maryam Haghighi‐Morad affirms that this manuscript is an honest, accurate, and transparent account of the study being reported; that no important aspects of the study have been omitted; and that any discrepancies from the study as planned have been explained.

## Ethics Statement

The study was approved by the Ethics Committee of Shahid Beheshti University of Medical Sciences (Ethics Code: IR.SBMU.MSP.REC.1403.069). Written informed consent was obtained from all participants before inclusion in the study. All procedures were conducted in accordance with the ethical standards of the institutional research committee and the principles of the Declaration of Helsinki and its later amendments.

## Conflicts of Interest

The authors declare no conflicts of interest.

## Data Availability

The data that support the findings of this study are available on request from the corresponding author. The data are not publicly available due to privacy or ethical restrictions.

## References

[1] K. McDermott , M. Fang , A. J. M. Boulton , E. Selvin , and C. W. Hicks , “Etiology, Epidemiology, and Disparities in the Burden of Diabetic Foot Ulcers,” Diabetes Care 46, no. 1 (2023): 209–221. 36548709 10.2337/dci22-0043 PMC9797649

[2] A. Johannesson , G. U. Larsson , N. Ramstrand , A. Turkiewicz , A. B. Wirehn , and I. Atroshi , “Incidence of Lower‐Limb Amputation in the Diabetic and Nondiabetic General Population: A 10‐Year Population‐Based Cohort Study of Initial Unilateral and Contralateral Amputations and Reamputations,” Diabetes Care 32, no. 2 (2009): 275–280. 19001192 10.2337/dc08-1639 PMC2628693

[3] D. G. Armstrong , A. J. M. Boulton , and S. A. Bus , “Diabetic Foot Ulcers and Their Recurrence,” New England Journal of Medicine 376, no. 24 (2017): 2367–2375. 28614678 10.1056/NEJMra1615439

[4] B. A. Lipsky , J. Aragon‐Sanchez , M. Diggle , et al., “IWGDF Guidance on the Diagnosis and Management of Foot Infections in Persons With Diabetes,” Diabetes/Metabolism Research and Reviews 32, no. Suppl 1 (2016): 45–74. 26386266 10.1002/dmrr.2699

[5] L. Giurato , M. Meloni , V. Izzo , and L. Uccioli , “Osteomyelitis in Diabetic Foot: A Comprehensive Overview,” World Journal of Diabetes 8, no. 4 (2017): 135–142. 28465790 10.4239/wjd.v8.i4.135 PMC5394733

[6] B. A. Lipsky , A. R. Berendt , H. G. Deery , et al., “Diagnosis and Treatment of Diabetic Foot Infections,” Clinical Infectious Diseases 39, no. 7 (2004): 885–910. 15472838 10.1086/424846

[7] A. R. Berendt , E. J. Peters , K. Bakker , et al., “Diabetic Foot Osteomyelitis: A Progress Report on Diagnosis and a Systematic Review of Treatment,” Diabetes/Metabolism Research and Reviews 24, no. Suppl 1 (2008): S145–S161. 18442163 10.1002/dmrr.836

[8] E. M. Senneville , B. A. Lipsky , S. A. V. van Asten , and E. J. Peters , “Diagnosing Diabetic Foot Osteomyelitis,” Diabetes/Metabolism Research and Reviews 36, no. Suppl 1 (2020): e3250. 31950555 10.1002/dmrr.3250

[9] G. M. Caputo , P. R. Cavanagh , J. S. Ulbrecht , G. W. Gibbons , and A. W. Karchmer , “Assessment and Management of Foot Disease in Patients With Diabetes,” New England Journal of Medicine 331, no. 13 (1994): 854–860. 7848417 10.1056/NEJM199409293311307

[10] B. A. Lipsky , A. R. Berendt , P. B. Cornia , et al., “2012 Infectious Diseases Society of America Clinical Practice Guideline for the Diagnosis and Treatment of Diabetic Foot Infections,” Clinical Infectious Diseases 54, no. 12 (2012): e132–e173. 22619242 10.1093/cid/cis346

[11] M. T. Dinh , C. L. Abad , and N. Safdar , “Diagnostic Accuracy of the Physical Examination and Imaging Tests for Osteomyelitis Underlying Diabetic Foot Ulcers: Meta‐Analysis,” Clinical Infectious Diseases 47, no. 4 (2008): 519–527. 18611152 10.1086/590011 PMC7450707

[12] J. La Fontaine , K. Bhavan , D. Jupiter , L. A. Lavery , and A. Chhabra , “Magnetic Resonance Imaging of Diabetic Foot Osteomyelitis: Imaging Accuracy in Biopsy‐Proven Disease,” Journal of Foot and Ankle Surgery 60, no. 1 (2021): 17–20. 10.1053/j.jfas.2020.02.012 33214100

[13] L. Pieruzzi , V. Napoli , C. Goretti , et al., “Ultrasound in the Modern Management of the Diabetic Foot Syndrome: A Multipurpose Versatile Toolkit,” International Journal of Lower Extremity Wounds 19, no. 4 (2020): 315–333. 32820699 10.1177/1534734620948351

[14] N. M. Buderer , “Statistical Methodology: I. Incorporating the Prevalence of Disease Into the Sample Size Calculation for Sensitivity and Specificity,” Academic Emergency Medicine 3, no. 9 (1996): 895–900. 8870764 10.1111/j.1553-2712.1996.tb03538.x

[15] M. Monteiro‐Soares , E. J. Hamilton , D. A. Russell , et al., “Guidelines on the Classification of Foot Ulcers in People With Diabetes (IWGDF 2023 Update),” Diabetes/Metabolism Research and Reviews 40, no. 3 (2024): e3648. 37179483 10.1002/dmrr.3648

[16] F. Gaillard , Y. Glick , L. Le , et al., “Osteomyelitis,” Radiopaedia.org (2009), accessed June 19, 2026, 10.53347/rID-7662.

[17] S. D. Wall , M. R. Fisher , E. G. Amparo , H. Hricak , and C. B. Higgins , “Magnetic Resonance Imaging in the Evaluation of Abscesses,” AJR. American Journal of Roentgenology 144, no. 6 (1985): 1217–1221. 3873805 10.2214/ajr.144.6.1217

[18] A. Rahmouni , O. Chosidow , D. Mathieu , et al., “MR Imaging in Acute Infectious Cellulitis,” Radiology 192, no. 2 (1994): 493–496. 8029421 10.1148/radiology.192.2.8029421

[19] É. Senneville , Z. Albalawi , S. A. van Asten , et al., “IWGDF/IDSA Guidelines on the Diagnosis and Treatment of Diabetes‐Related Foot Infections (IWGDF/IDSA 2023),” Diabetes/Metabolism Research and Reviews 40, no. 3 (2024): e3687. 37779323 10.1002/dmrr.3687

[20] M. D. Enderle , S. Coerper , H. P. Schweizer , et al., “Correlation of Imaging Techniques to Histopathology in Patients With Diabetic Foot Syndrome and Clinical Suspicion of Chronic Osteomyelitis. The Role of High‐Resolution Ultrasound,” Diabetes Care 22, no. 2 (1999): 294–299. 10333948 10.2337/diacare.22.2.294

[21] C. Lauri , E. Noriega‐Álvarez , R. M. Chakravartty , et al., “Diagnostic Imaging of the Diabetic Foot: An EANM Evidence‐Based Guidance,” European Journal of Nuclear Medicine and Molecular Imaging 51, no. 8 (2024): 2229–2246. 38532027 10.1007/s00259-024-06693-y PMC11178575

[22] H. H. Alsararatee , J. C. S. Langley , M. Thorburn , H. Burton‐Gow , S. Whitby , and S. Powell , “Assessment of the Diabetic Foot in Inpatients,” British Journal of Nursing 34, no. 4 (2025): S12–S23. 10.12968/bjon.2024.0342 39969836

[23] K. Daneshvar and H. Anwander , “Diagnostic Imaging of Diabetic Foot Disorders,” Foot and Ankle Clinics 27, no. 3 (2022): 513–527. 36096549 10.1016/j.fcl.2022.01.002

[24] A. Komarraju and A. Chhabra , “Advanced Cross‐Sectional Radiology—Ultrasound, Computed Tomography and Magnetic Resonance Imaging of the Diabetic Foot,” In The Foot in Diabetes 5th ed. (Wiley, 2020), 169–185, 10.1002/9781119445821.ch10.

[25] S. Adhikari and M. Blaivas , “Sonography First for Subcutaneous Abscess and Cellulitis Evaluation,” Journal of Ultrasound in Medicine 31, no. 10 (2012): 1509–1512. 23011612 10.7863/jum.2012.31.10.1509

[26] L. K. Horton , J. A. Jacobson , A. Powell , D. P. Fessell , and C. W. Hayes , “Sonography and Radiography of Soft‐Tissue Foreign Bodies,” AJR. American Journal of Roentgenology 176, no. 5 (2001): 1155–1159. 11312171 10.2214/ajr.176.5.1761155

[27] X. Wang , W. Zhang , J. Dong , L. Li , Y. Xiao , and F. Liu , “Three‐Dimensional Sonography Has Satisfied Accuracy for Detecting Rotator Cuff Tears,” Frontiers in Surgery 11 (2024): 1411816. 38812755 10.3389/fsurg.2024.1411816 PMC11133732

[28] S. Subramaniam , J. Bober , J. Chao , and S. Zehtabchi , “Point‐Of‐Care Ultrasound for Diagnosis of Abscess in Skin and Soft Tissue Infections,” Academic Emergency Medicine 23, no. 11 (2016): 1298–1306. 27770490 10.1111/acem.13049

[29] P. Spinnato , D. B. Patel , M. Di Carlo , et al., “Imaging of Musculoskeletal Soft‐Tissue Infections in Clinical Practice: A Comprehensive Updated Review,” Microorganisms 10, no. 12 (2022): 2329. 36557582 10.3390/microorganisms10122329 PMC9784663

[30] E. F. Alaia , A. Chhabra , C. S. Simpfendorfer , et al., “MRI Nomenclature for Musculoskeletal Infection,” Skeletal Radiology 50, no. 12 (2021): 2319–2347. 34145466 10.1007/s00256-021-03807-7 PMC8789645

[31] S. Kaiser and M. Rosenborg , “Early Detection of Subperiosteal Abscesses by Ultrasonography. A Means for Further Successful Treatment in Pediatric Osteomyelitis,” Pediatric Radiology 24, no. 5 (1994): 336–339. 7824366 10.1007/BF02012120

[32] J. M. Mellado Santos , “Diagnostic Imaging of Pediatric Hematogenous Osteomyelitis: Lessons Learned From a Multi‐Modality Approach,” European Radiology 16, no. 9 (2006): 2109–2119. 16541223 10.1007/s00330-006-0187-4

[33] N. B. Wright , G. T. Abbott , and H. M. Carty , “Ultrasound in Children With Osteomyelitis,” Clinical Radiology 50, no. 9 (1995): 623–627. 7554737 10.1016/s0009-9260(05)83292-1

[34] T. Hosokawa , K. Deguchi , H. Takei , Y. Sato , Y. Tanami , and E. Oguma , “Ultrasonography for the Detection of Osteomyelitis in Pediatric Patients With Soft Tissue Infection: A Pilot Study,” Journal of Ultrasound in Medicine 43, no. 7 (2024): 1223–1234. 38456324 10.1002/jum.16446

[35] T. W. Riebel , R. Nasir , and O. Nazarenko , “The Value of Sonography in the Detection of Osteomyelitis,” Pediatric Radiology 26, no. 4 (1996): 291–297. 8677150 10.1007/BF01372116

[36] C. B. Howard , M. Einhorn , R. Dagan , and M. Nyska , “Ultrasound in Diagnosis and Management of Acute Haematogenous Osteomyelitis in Children,” Journal of Bone and Joint Surgery. British Volume 75, no. 1 (1993): 79–82. 8421042 10.1302/0301-620X.75B1.8421042

[37] T. Greco , A. Mascio , C. Comisi , et al., “RANKL‐RANK‐OPG Pathway in Charcot Diabetic Foot: Pathophysiology and Clinical‐Therapeutic Implications,” International Journal of Molecular Sciences 24, no. 3 (2023): 3014. 36769345 10.3390/ijms24033014 PMC9917950

